# On the Trail of Viroids a Return to Phytosanitary Awareness

**DOI:** 10.3390/pathogens14060545

**Published:** 2025-05-29

**Authors:** Moshe Bar-Joseph

**Affiliations:** The S. Tolkowsky Laboratory, Department of Plant Pathology and Weed Research, Agricultural Research Organization—The Volcani Center, P.O. Box 15159, Rishon Lezion 7528809, Israel; mbjoseph@gmail.com

**Keywords:** history of grafting, plant disease emergence, pathogen-induced dwarfing, acquired resistance, high-density planting, diagnostics

## Abstract

Viroids are subviral plant pathogens composed of non-coding, small, circular, single-stranded RNAs that parasitize the transcriptional machinery of their host cells. For many years, viroid-induced diseases were mistakenly attributed to viruses due to similarities in symptoms and pathogenic behavior. However, advances in molecular biology over the past sixty years have clearly distinguished viroids from viruses and other pathogens in terms of genetic composition, structural features, and replication mechanisms. Citrus trees in the Mediterranean region appear to have been associated with viroid infections since ancient times. Nevertheless, the use of propagation material harboring asymptomatic viroid infections allowed for continued production of high-quality fruit. This delicate equilibrium was disrupted with the spread of novel citrus pathogens, prompting the adoption of new horticultural practices that emphasized the elimination of citrus pathogens—including viroids—from propagation material. Concurrently, a contrasting approach emerged in the late 1960s: the experimental use of “graft-transmissible dwarfing agents”—later identified as citrus viroids—to control citrus tree size. Our lab initiated work on citrus viroid-induced dwarfing in the early 1980s and continued this line of research for nearly two decades. Eventually, we concluded that it was impractical to simultaneously promote rigorous sanitation protocols while advocating for the use of viroids to induce dwarfing. This review summarizes key biological and molecular aspects of citrus and avocado viroids investigated in our laboratory, including the development of diagnostic techniques and the exploration of viroid-induced dwarfing as a horticultural tool.

## 1. Introduction

Viroids [1,2,3,4,5,6,7,8,9,10] are small, circular, single-stranded, RNA pathogens that parasitize the transcriptional machinery of infected plant cells. Due to their subviral size and lack of protein-coding capacity, viroid-associated diseases were historically misattributed to elusive viral infections. Over the past six decades, advances in biophysical methods and nucleotide sequence analysis have elucidated the unique molecular features of viroid genomes, enabling their clear differentiation from viruses and other plant pathogens.

The foundational insights of viroid biology were gained from studies on potato spindle tuber viroid (PSTVd) [11,12,13,14,15] and citrus exocortis [16,17,18,19], a common old-clone citrus disease [20] of complex etiology [21,22] caused by citrus exocortis viroid (CEVd) [18,19] in conjunction with other citrus viroids [21,22,23,24,25,26].

Traditionally, Mediterranean fruit trees—including citrus, grapevines, pomes, and stone fruits—were mainly propagated using cuttings or as non-grafted seedlings [27]. The small orchards were often densely planted in mixed groves, facilitating the dissemination of multiple viroid species, which however were seldom associated with significant crop damage. This changed with horticultural modernization, including the introduction and transfer of budwood as propagation material, and rooted plants. Replacing the much safer method of importing new citrus varieties through seeds. This shift resulted in the introduction of exotic trees and shrubs infected with soil borne pathogens and viruses, contributing to the emergence of severe disease complexes, such as gummosis, root rot, and the tristeza virus epidemic [28].

These crises prompted a transition from non-grafted trees to stionic combinations with rootstocks resistant or tolerant to root rot and, later, to tristeza. However, this transition introduced new challenges: the novel scion–rootstock combinations were often poorly adapted to endemic viroid populations that had co-evolved with traditional, long-established citrus clones [27,28].

The realization that seedlings derived from polyembryonic seeds of exocortis-infected trees were free of exocortis [20] and xyloporosis [29] diseases enabled the production of true-to-type, disease-free nucellar trees [30]. This development allowed for more accurate assessment of the exocortis pathogenicity on sensitive rootstocks, including trifoliate orange, Rangpur lime, and sweet lime. These tests demonstrated that budwood infected with exocortis caused significantly reduced tree size on certain sensitive rootstocks, particularly trifoliate orange [31].

A similar adaptation challenge occurred during the transition from vegetative propagation of rootstocks to propagation from seed. When exocortis-free budwood was grafted onto seedling rootstocks of Sweet lime and Rangpur lime, the resulting trees showed increased susceptibility to Phytophthora-induced root rot [32]. This vulnerability could only be mitigated by inarching with sour orange rootstocks, which are known for their resistance.

While empirical practices helped sustain affected orchards, the molecular basis of these complex disease syndromes remained poorly understood until the 1970s. At that time, advances in viroid biology began to elucidate the nature of the exocortis disease complex. The recognition of the physiological disorders caused by the use of latent exocortis infections, led to global efforts aimed at detecting and eliminating this and similar disease agents from citrus and other vegetatively propagated plant materials. These efforts were supported by new methods for pathogens elimination from budwood sources [33] and by the implementation of phytosanitary measures and certification programs designed to prevent the spread of viroid infections through propagation material [34].

However, a contrasting perspective—held by a minority within the citrus virology community—questioned the blanket assumption that all pathogens are necessarily detrimental. This alternative view proposed that pathogens should be assessed based on their net agronomic impact, particularly in terms of yield quantity and quality, rather than their mere presence [31]. Theoretical models suggested that yield losses in individual viroid-affected trees could be offset by high-density plantings, resulting in comparable overall yields and the additional benefit of smaller, more manageable trees.

It was within this context that the concept of using exocortis diseased budwood as biological dwarfing agents began to take shape in the late 1960s. This unorthodox approach involved the deliberate use of “graft-transmissible dwarfing agents”—later identified as specific citrus viroids—to control tree vigor and optimize orchard architecture.

Our own work on citrus viroid-induced dwarfing began in the 1980s and continued for nearly two decades. The research aimed to harness the growth-regulating properties of certain citrus viroid complexes to produce compact, productive citrus trees suited for high-density plantings. Despite initial promise and considerable interest, the approach remained controversial and ultimately fell out of favor. My personal engagement with promoting viroid-based dwarfing strategies came to an end—reminiscent of a retreat to Canossa.

Two main factors contributed to the abandonment of the viroid dwarfing program and the renewed emphasis on phytosanitary awareness, including:

1. The shift in Israeli citriculture to the southern parts of the country, where soils were less suitable for viroid-sensitive rootstocks and higher salinity levels proved especially damaging to viroid-infected trees;
2. Changes in local citrus management practices revealed that recommendations advocating viroid inoculation for dwarf tree production often overlooked or disregarded phytosanitation guidelines aimed at preventing the spread of viroids in sensitive scion-rootstock combinations. This was evidenced by increased incidences of poor performance in trees grafted onto viroid-sensitive rootstocks, such as Troyer citrange. Ultimately, we concluded that it was impractical to simultaneously promote viroid-induced dwarfing and emphasize proper sanitation.

This paper follows a recent publication on Citrus tristeza virus (CTV) [35]—the primary focus of my research for much of my career. My work in plant virology began shortly before the term *viroid* was formally introduced and has extended well into the molecular era. This review offers a personal, professional perspective shaped by decades of service work and research on diseases of citrus and avocado trees caused by viroids. As such, the article includes numerous self-citations reflecting the trajectory of my own scientific work. However, this emphasis should not be interpreted as a measure of the relative importance of the cited works. On the contrary, most foundational and contemporary papers that significantly advanced the field of viroid research cited in reviews [1,2,3,4,5,6,7,8,9,10] are only marginally cited herein. This is due mainly to the personalized nature of this retrospective study. The omission of many pivotal contributions by colleagues and collaborators is acknowledged with deep appreciation for their indispensable role in shaping our current understanding of viroid biology and pathology.

## 2. The Pre-Viroid Period

### 2.1. Avocado Sunblotch Disease

My work at the Virus Laboratory, Volcani Center, Israel, was partially funded by a PL480 grant aimed at investigating whether free-living nematodes could transmit avocado sunblotch disease (ASBd). We collected soil samples from around symptomatic avocado trees (cv. Lula, West Indian) at Mikveh Yisrael—the first agricultural high school in Israel and also my alma mater. The soil, potentially containing free-living nematodes, was placed in 50 L containers with healthy cv. Hass avocado seedlings were planted and maintained in an insect-proof screenhouse.

At the time, free-living nematodes were known vectors of certain plant viruses [36], and ASBd was assumed to be virus-induced due to its graft transmissibility and the absence of a cultivable pathogen. The etiology of avocado sunblotch viroid (ASBVd) and its sequence were identified in the years 1979 [37] and 1981 [38], respectively. Thus, my early work on ASBd and citrus exocortis predated the discovery of free RNAs as pathogens [11,12,16] and the coining of the new term “*viroid*” for these unique pathogens [13].

### 2.2. Unexpected Findings from Failed Nematode Transmission Experiments

Despite maintaining the Hass seedlings in nematode-infested soil for over five years, none developed ASBd symptoms. Although the experiments failed to demonstrate nematode transmission, they led to unexpected insights.

Two to three years after planting, some cv Hass avocado trees developed deep necrotic pits on their suberized stems. Given that citrus plants infected with Citrus tristeza virus (CTV) were housed in the same screenhouse, we initially suspected CTV contamination. However, the stem pitting was later attributed to Latania scale (*Hemiberlesia lataniae*) [39].

A separate virus-like disease of avocado—marked by severe leaf deformation and mottling, and seemingly graft-transmissible—that we traced to an infestation of broad mites (*Polyphagotarsonemus latus*) represents the first report of this serious pest in Israel [40].

### 2.3. Citrus Exocortis Disease

My second assignment focused on the biological indexing of citrus propagation material for virus diseases, including exocortis [41]. Citron cuttings and seedlings were used as indicator plants. However, symptom expression on these plants varied greatly—some showed pronounced epinasty, while others exhibited only mild symptoms. Attempts to enhance epinasty intensity using hormone treatments were inconclusive and eventually abandoned.

The wide variability in exocortis symptoms among genetically identical cuttings remained puzzling until it was discovered that old citrus budwood clones were often infected with multiple citrus viroid species, each differing in pathogenicity on Etrog indicator plants [24]. My indexing work on introduced citrus varieties continued until the natural spread of CTV became apparent, prompting the launch of a national CTV suppression program [35] and a shift in my research focus.

### 2.4. Molecular Diagnosis of Viroids

In the early 1980s, we began applying molecular hybridization techniques to differentiate certain CTV strains [42] and to replace the lengthy ASBVd biological indexing procedures [43]. The primary goal was to identify and eliminate trees infected with seed-transmissible latent (STL) ASBVd isolates [44]. Surprisingly, symptomless ASBVd-infected trees harbored large quantities of viroid RNA. We separated and [^32^P]-labeled these molecules for use as hybridization probes, improving detection sensitivity by 16- to 64-fold over traditional PAGE analysis [43].

Later, we developed radioactively labeled oligonucleotide probes to detect ASBVd in crude and partially purified RNA extracts [45]. These probes proved especially useful for identifying exotic pathogens prior to their introduction and spread. Interestingly, while oligonucleotide probes consistently detected ASBVd in symptomless STL infections, detection in symptomatic trees was more erratic. This underscored the importance of combining visual assessment with multi-site tissue sampling for reliable mother tree diagnosis [46].

### 2.5. The Shift Toward Using Citrus Viroids for Dwarfing

For an excellent horticultural overview of viroid dwarfing strategies, readers are referred to [31]. In Israel, the concept of using “graft-transmissible dwarfing agents” to regulate citrus tree size was first proposed by Mendel [47,48], then head of the Citriculture Department. Mendel observed that old-clone grapefruit trees on trifoliate orange (*Poncirus trifoliata*) rootstock, infected with exocortis (then presumed viral), were significantly smaller and began bearing fruit one to two years earlier than trees grafted with viroid-free tissue. He posited that high-density planting (HDP) of such dwarfed trees could yield equal or greater productivity compared to larger, healthy trees.

A second factor sustaining interest in viroid-infected budwood was the early decline—sometimes occurring within 1–2 years—of trees on Rangpur lime or sweet lime rootstocks grafted with viroid-free material (Oren Y, personal communication). In contrast, trees grafted with the old-clone, exocortis-infected budwood remained productive, with reduced size and consistent fruit quality. The protective effect was attributed to exocortis-induced tolerance against soil borne pathogens [49] to which these rootstocks were particularly sensitive.

Despite this, efforts to implement Mendel’s approach faced setbacks due to considerable variability in tree size among trees grafted with the GTD-225 dwarfing budwood source [50]. This inconsistency was linked to the uneven distribution of different exocortis viroid isolates within source trees.

Dwarfing trials continued at a small scale in the 1980s [50,51,52], when the late Amitay Rasis—a citrus extension specialist—inspired by Australian work [31,53,54], championed a large-scale dwarfing program. Rasis envisioned that dwarf citrus trees would resolve an ideological challenge in kibbutz farming, where seasonal labor was at odds with collective labor ideals. Dwarf trees, being easier to harvest, would allow elderly members to participate in the harvest. However, phytosanitary restrictions on importing Australian viroids required localized research instead.

### 2.6. Biological and Biophysical Assays of the GTD-225 Complex

Under Rasis’ initiative, our key objective was to investigate the molecular basis for the wide variation in grapefruit tree size observed in trees grafted with GTD-225-T, budwood on trifoliate rootstocks. A small experimental orchard established at Newe Yaar enabled us to categorize trees into very mild (VM), mild (M), and severe (S) dwarfing subtypes. When Etrog citron plants were inoculated with budwood from these subtypes, VM and M induced only mild leaf epinasty, whereas S induced severe epinasty and stunting [50,55].

### 2.7. Sequential PAGE Analyses of Citrus Viroids in GTD-225

Etrog citron plants inoculated with GTD-225-T or GTD-225-S grapefruit budwood displayed five viroid bands when analyzed via sequential PAGE (sPAGE) for circular RNAs. These included the well-characterized 371-nucleotide citrus exocortis viroid (CEVd) and four additional viroids measuring ~330, 300, 295, and 275 nucleotides.

In contrast, the VM and M subtypes lacked the CEVd band and contained only three viroids: ~330, 295, and 275 nucleotides in the M subtype, and ~300, 295, and 275 in the VM subtype [24].

These findings indicated that variation in dwarfing severity in GTD-225-inoculated trees was due to differential infection with the full citrus viroid (CVd) complex present in the original GTD-225-T source. Interestingly, while the viroid composition varied in grapefruit trees, it remained stable in Etrog citron plants, reproducibly inducing characteristic symptoms and viroid profiles through three successive transfers. The primary takeaway from these experiments was that, for consistent dwarfing outcomes, viroid inoculum should be propagated from Etrog citron tissue rather than directly from grapefruit, ensuring uniform viroid profiles across inoculated trees.

## 3. Sequencing the GTD-225 Citrus Viroids

### 3.1. Characterization of the HSVd Component in GTD-225

A collaborative grant with Dr. H.L. Sänger’s group led to the molecular characterization of two viroids from the GTD-225 complex. The first was isolated by propagation in *Benincasa hispida*, a cucurbit host in which it caused severe leaf malformation and stunting—symptoms typical of Hop stunt viroid (HSVd). The HSVd–grapefruit isolate consisted of 299 nucleotides [56] and differed from the Japanese HSVd type strain (297 nts) at seven nucleotide positions [57]. It was considered a potential intermediate between the type of strain and the cucumber pale fruit HSVd isolate.

Both GTD-225 and the cv Shamouty-Nir Galim old-clone, termed GTD–NG, often used for grafting commercially on Rangpur lime, were found to contain not only the HSVd isolate sequenced by Puchta et al. [57] but also a cachexia–Xyloporosis-inducing HSVd variant [32]. This more pathogenic variant was apparently attenuated by the non-cachexia HSVd type; however, the degree of attenuation varied. Some cases exhibited cachexia–Xyloporosis symptoms, contributing to the mistaken belief that unauthorized GTD sources were responsible for the variable dwarfing outcomes.

### 3.2. Characterization of CVd-IV (Citrus Bark Cracking Viroid)

The second viroid from the GTD-225 complex, characterized by Sänger’s group, was CVd-IV, now known as citrus bark cracking viroid (CBCVd). CVd-IV [58], consists of 284 nucleotides arranged in a viroid-specific rod-like secondary structure, with 71% of its nucleotides being base-paired. Initially proposed to belong to the genus *Apscaviroid*, it is now classified under the family *Pospiviroidae*, genus *Cocadviroid*, based on its possession of a terminal conserved hairpin (TCH) and absence of a terminal conserved region (TCR)—structural features typical of *Cocadviroid* members [59].

### 3.3. Further CVd Sequence Analyses

Ben-Shaul et al. [60] examined the genomes of Citrus exocortis viroid (CEVd) variants from various local GTD sources. Five CEVd variants from four GTDs showed high similarity (2–9 nucleotide differences), while five others from two different GTDs displayed greater divergence (27–50 nucleotide differences). The most significant variability was found within a single source, GTD-G, with differences of up to 41 nucleotides.

Using GTD-225-derived CEVd as a reference, most mutations were localized to the V, LT, and RT domains, with additional changes in the P domain. These were particularly prevalent in isolates from GTD-M and GTD-G—both highly aggressive on sensitive tomato cultivars. Sequence homologies to CEVd-W225 ranged from 89.2% (CEVd-G) to 99.0% (CEVd-NG). Notably, GTD-NG is the only complex still recommended for dwarfing sweet orange cv. Shamouty on Rangpur lime under optimal soil conditions.

### 3.4. Isolation and Characterization of Citrus Bent Leaf Viroid (CBLVd)

Grafting GTD-225-infected citrus buds onto avocado seedlings enabled the transfer and isolation of CVd-I from the viroid complex [61]. This finding facilitated the biological and molecular characterization of a viroid similar to CV-Ib, later named Citrus-bent leaf viroid (CBLVd). CBLVd was isolated from avocado leaves, and its circular RNA molecules were polyadenylated, reverse transcribed using a chimeric polylinker-oligo dT (P-dT) primer, and cloned into the pBluescript KS(+) vector. Positive inserts were sequenced using the Sequenase^®^ protocol [62].

CBLVd comprises 318 nucleotides, forming a rod-like viroid-specific structure with more than 66% of bases paired. Its molecular size, smaller than the previously estimated 330 nts (via sPAGE), confirmed it as a novel viroid and the first *Apscaviroid* species-infecting citrus [63].

Sequence analysis of CBLVd variants [60] revealed limited variation (0–7 nts) within a single GTD source and greater divergence (8–15 nts) among different sources. Notably, the avocado-derived type strain differed at six nucleotide positions (38, 62, 138, 179, 264, and 268), suggesting host-dependent sequence adaptation—a phenomenon previously observed in other viroids [64].

Attempts to use CBLVd to dwarf avocado trees in a five-year insect-proof containment trial were unsuccessful, as all inoculated trees remained symptomless [61].

### 3.5. Ultra High-Density Planting (HDP) Experiment in the Hulla Valley

A large-scale HDP experiment tested citrus dwarfing at tree spacings of 1000 and 1660 trees/hectare, compared to the standard 400 trees/hectare control. Four commercial cultivars—Oroblanco, Star Ruby, Minneola, and Ortanique—were grafted onto both local and Californian Benikke trifoliate rootstocks. Two GTD sources, #225-S (Etrog-derived) and GTD #BD (CVd-II and CVd-III), were inoculated in the field at 8 and 20 months post-planting.

Initial yields showed marked increases under high-density planting. For instance, Oroblanco trees planted at higher densities yielded double those planted conventionally [65]. Interestingly, while GTD-225-inoculated Star Ruby trees showed pronounced dwarfing and bark scaling 3–4 years post-inoculation, similarly treated Oroblanco trees on trifoliate rootstocks did not. Five to seven years post-planting, differences became evident: the GTD-225-topped Oroblanco trees produced shorter shoots (30–60 cm) compared to over 1 m shoots on the untreated controls. These findings suggested a delayed dwarfing effect in Oroblanco under optimal conditions.

Unfortunately, with the retirement of Mr. Rasis and diminished interest and continuation of this research, particularly as similar commercial plantings on Troyer citrange rootstocks on less suitable soils produced poorly, our work on GTD-based dwarfing came to a halt.

### 3.6. Acquired Tolerance to Malsecco Through Citrus Viroid Inoculation

Inspired by Dr. Vittoria Rosetti’s discovery [49] of Phytophthora root rot tolerance in exocortis-infected citrus rootstocks, we explored whether CEVd pre-inoculation could confer tolerance to *Phoma tracheiphila*, the causal agent of Malsecco.

Etrog citron plants inoculated with the GTD-225-derived CEVd developed characteristic viroid symptoms (leaf curling, shortened internodes). One year post-inoculation, plants were challenged with the Malsecco pathogen. While leaf lesions appeared in both infected and control plants, pathogen spread was significantly reduced in viroid-inoculated Etrog citron: only 9.1% of branches tested positive compared to 100% in controls. In Rangpur lime, infection rates ranged from 16.7% to 50% in viroid-treated plants, compared to 70% in controls [55].

Follow-up studies in CEVd-tolerant lemon cultivars showed no significant Malsecco protection [Bar-Joseph, Unpublished], suggesting that tolerance induction is limited to hosts that exhibit viroid symptoms, rather than merely supporting CVd replication.

### 3.7. Movement of CEVd in Etrog Citron Seedlings

CEVd distribution post-inoculation was monitored in root and stem tissues over time. Initial accumulation was observed in basal and root tissues, later spreading to shoots. Interestingly, CEVd RNA migrated toward roots at a rate apparently similar to the much larger citrus tristeza virus (CTV) virions. However, the timing of the passage of these two different size and composition pathogens was not assayed on a daily basis, and differences may be found with higher temporal resolution [66].

## 4. Effects of Viroids on Citrus Water Relations

### 4.1. Greenhouse Tests

Due to drought-related citrus acreage reductions in Israel, in the 1980s, we investigated CEVd effects on plant water dynamics. CEVd-infected Etrog citron seedlings were subjected to drying cycles. Compared to controls, infected plants had 16% less leaf area (*p* < 0.05), 10% lower midday water uptake (*p* < 0.001), and decreased leaf conductance, although total hydraulic conductance was unchanged, likely due to altered cell wall properties or root-derived stress signals [67,68].

### 4.2. Field Tests

In a drip-irrigated grapefruit grove (Star Ruby on Troyer Citrange), viroid-infected trees under wet and dry irrigation were monitored. Water uptake, leaf conductance, and stem water potential were consistently lower in infected trees—especially noticeable from the sixth year post-inoculation. Again, such reductions were associated with the symptomatic phase of viroid infections [69].

### 4.3. Goats as Vectors of Viroids

Mechanical inoculation and infected budwood propagation were long thought to be the primary routes of viroid spread. However, these methods cannot explain viroid presence in graft-incompatible or remote wild trees. RNA extracted from goat (*Capra hircus*) horns that had been rubbed against viroid-infected Etrog stems contained both CEVd and HSVd. Transmission was confirmed by rubbing healthy citrus plants with contaminated horns 24 h later. These results suggest that goats, herded for many millennia in the region, could have contributed to the wide range of viroids among ancient Mediterranean trees and vines [70].

### 4.4. Multiprobe for Simultaneous Detection of Citrus Viroids

After phasing out the GTD approach, we focused on viroid detection methods. A CVd-Multiprobe composed of full-length HSVd, CEVd, CBLVd, and CVd-III clones was created and tested. The DIG-labeled probe successfully detected infections using northern and dot blot hybridization. Although the probe cannot differentiate between individual CVds, this limitation is acceptable for most certification programs, which require the removal of any infected budwood source regardless of viroid type. Its simplicity and efficacy made the multiprobe suitable for routine diagnostics [71].

## 5. The Failures

Throughout my scientific career, my curiosity and drive to test new ideas far outpaced the number of papers we ultimately published. This was largely because failures outnumbered fruitful outcomes by a factor of ten. I have often questioned whether withholding the results of failed experiments is scientifically justified. In many cases, such data could have provided valuable insights—not only by identifying unproductive paths but also by suggesting alternative directions worth pursuing.

For example, as noted earlier, we initially failed to resolve the variation in epinasty observed in Etrog citron plants inoculated with different exocortis budwood sources. It took more than a decade to begin understanding the underlying causes of this variation [21,22].

In my attempts to purify Citrus tristeza virus (CTV) particles, we used tissue infected with a mixture of CTV, exocortis, and xyloporosis. At the time, xyloporosis was considered a serious threat to trees grafted on sweet lime, and I was repeatedly encouraged by my superiors to isolate its causal agent [32]. Unfortunately, I was never able to follow through on this directive—none of the many particle isolation efforts yielded any virus-like particles other than the thread-like forms of CTV. No viral particles appeared to be associated with either xyloporosis or exocortis infections [35].

When exploring the origins of citrus viroids (CVds), we once speculated that the Etrog fruit deformation known as *gartel*—often depicted in ancient mosaics from the 3rd to 6th centuries CE—might be historical evidence of CVd infections. However, Schaffer A. (personal communication]) later clarified that *gartel* also occurs in CEVd-free Etrog fruit and is caused by girdling from petal coral residue on young fruits. Nonetheless, the possible spread of CVd via “goat-horn” contact remains a plausible indicator that viroids were present in the region even before the era depicted in those mosaics.

Other unreported failures include a two-year PhD project that aimed to unravel the genetic basis of CEVd-induced epinasty. Using Dr. D. Zamir’s tomato introgression lines [72], we identified chromosomal regions associated with the trait and were close to publishing our first paper on the “epinasty locus” in tomato. However, we eventually discovered that the trait was not governed by a single locus. Confronted with this complexity, we discontinued the study to allow the student to graduate on time.

To this day, the epinasty syndrome remains unresolved, as do the mechanisms behind bark cracking and dwarfing—phenomena likely linked to viroid RNA’s regulatory activity. Other significant unanswered questions include the factors influencing the efficacy of cross-protection between HSVd isolates.

## 6. Discussion

Two landmark reports in 1967 [11,73] fundamentally altered the course of plant virology. Doi and colleagues [73] described a new class of plant pathogens: mycoplasma-like organisms restricted to the phloem of yellowing plants. Simultaneously, Diener and Raymer [11] reported that the agent responsible for potato spindle tuber disease was a free RNA molecule, distinct from conventional viruses. Not long afterward, similar findings were made concerning the agent of citrus exocortis disease [16].

Diener later showed that these RNA molecules represented a new class of plant pathogens, ultimately termed viroids [13]. The subsequent discovery and characterization of many viroid-induced diseases attracted attention from a wide range of scientists—not only plant pathologists and horticulturists but also microbiologists, molecular biologists, and biotechnologists intrigued by the viroids’ unique properties [1,2,3,4,5,6,7,8,9,10]. This review, written from a personal perspective, is a tribute to the pioneers who transformed traditional plant virology and to those who have continued to advance the field.

My first viroid project, on avocado sunblotch viroid (ASBVd), aimed to investigate soil transmission, a hypothesis that was eventually disproven. Natural transmission was later shown to occur via airborne pollen [74].

In citrus, I contributed to biological indexing, evaluating imported and local cultivars as potential replacements for declining Shamouti groves. My interest in citrus viroid-induced dwarfing was sparked in the 1980s by Mr. Amitay Rasis’ enthusiasm from promising trials in Australia [31,53,54,75]. High-density plantings in the Hula Valley initially produced impressive yields [64], earning the phenomenon the nickname “Hula Valley magic”. However, long-term results were disappointing. Incompatibility with rootstocks, poor soil conditions, and suboptimal irrigation water in newer growing areas led to tree decline. These problems were compounded by changing management practices and a gradual erosion of technical expertise. Ultimately, efforts to balance viroid-induced dwarfing with the need for sanitation proved unsustainable. Even in more structured programs in Australia, Italy, South Africa, and California, large-scale application of viroid-induced dwarfing remained limited.

A BARD-funded collaboration with Joe Semancik enabled us to investigate GTD-225-induced dwarfing. Dr. R. Hadas demonstrated that tree size variation correlated with the composition of the CVd population, providing the first direct evidence of viroid population segregation in certain citrus varieties [24].

ASBVd was eventually eradicated from local orchards through visual inspections and multi-site molecular sampling [46]. However, unlike ASBVd, citrus viroids (CVds) have persisted. Their endurance is partly due to their tolerance on preferred rootstocks like sour orange and their required retention in scions grafted onto root-rot-sensitive stocks. The introduction of sequential PAGE (sPAGE) and, later, DIG-labeled CVd multiprobes allowed us to scale up screening for CVd [72] and other viroid infections.

Alongside these successes came further disappointments. We investigated CVd-induced acquired immunity against the Malsecco fungus, but the protective effect was confined to symptomatic Etrog citron plants [66], which have no commercial value. Similarly, the observed tolerance to water stress was limited to these symptomatic citrons [67]. While this may explain their survival under historical Mediterranean drought conditions, it offered no modern agricultural benefits. Attempts to transfer CBLVd to avocado trees also failed to yield dwarfing or other desirable traits [61].

Our detection of CVd RNA on goat horns, along with successful transmission via stem rubbing [70], led us to hypothesize that traditional goat herding practices may have contributed to the widespread dissemination of viroids across the Mediterranean basin in ancient fruit tree clones.

In conclusion, our viroid research began with the hope of improving crop performance by leveraging pathogen-induced traits. The early results—especially in the favorable conditions of the Hula Valley—were encouraging. However, broader implementation proved elusive. Environmental constraints, shifting agronomic practices, and the biological complexity of viroids ultimately limited our success. Yet, as Prof. Ricardo Flores [76] wrote to me in 2009, viroids remain deeply fascinating—not only as plant pathogens but also as models for exploring the origins of life.

My journey along the viroid trail has been shaped by the advances of a dedicated group of researchers [1,2,3,4,5,6,7,8,9,10]. It has also served as a personal lesson in the caution required when attempting to repurpose a pathogen as a tool.

## Data Availability

No new data were created or analyzed in this study.
